# Late Complications Following Continuous-Flow Left Ventricular Assist Device Implantation

**DOI:** 10.3389/fsurg.2015.00042

**Published:** 2015-08-19

**Authors:** Joshua C. Grimm, J. Trent Magruder, Clinton D. Kemp, Ashish S. Shah

**Affiliations:** ^1^Division of Cardiac Surgery, The Johns Hopkins Hospital, Baltimore, MD, USA

**Keywords:** left ventricular assist device, late complications

## Abstract

Left ventricular assist devices have become standard therapy for patients with end-stage heart failure. They represent potential long-term solutions for a growing public health problem. However, initial enthusiasm for this technology has been tempered by challenges posed by long-term support. This review examines these challenges and out current understanding of their etiologies.

## Introduction

Technological advances have allowed for the emergence of newer generation, continuous-flow left ventricular assist devices (LVADs) approved for both bridge-to-transplant (BTT) as well as destination therapy (DT) in patients with end-stage heart failure. As outlined in the Third INTERMACS Annual Report, there has been a recent trend toward a greater number of devices being implanted in patients with an initial strategy of DT support (1). The Sixth INTERMACS Annual Report quantified further and demonstrated a nearly three-fold increase from 2006–2007 to 2011–2013 (14.7 and 41.6%, respectively) (2). Within this cohort of patients implanted between 2006 and 2010, approximately 80% were deemed INTERMACS level 2 (progressive decline), 3 (stable but inotrope-dependent), or 4 (recurrent, advanced heart failure) (1, 3), and as they did not represent “crash and burn” patients (INTERMACS level 1), expected short-term survivals were improved (4, 5).

Accordingly, while early complications following LVAD implantation have been extensively described and have been shown to greatly impact perioperative morbidity and mortality (6–8), it is reasonable to expect that the influence of late adverse events will be increasingly more relevant given the changing landscape of these implantable circulatory support devices. The following review will explore the long term, systemic effects of prolonged, continuous-flow LVAD support (device failures, bleeding, right ventricular dysfunction, infection, aortic valve pathology, and thromboembolic events) and briefly describe the most common management strategies employed in each scenario.

## Technical, Device Failures

Failure of modern, continuous-flow LVADs is exceedingly rare but can involve any component of the device (i.e., pump, controller, or power source). It is critically important that an emergency card is visible at all times when a patient is discharged to home with a LVAD. This will provide invaluable information to first responders in cases of device failure. More specifically, problems with the power source can typically be mitigated by a VAD coordinator or outpatient nurse as they are easily exchangeable without the need for an invasive procedure.

If, however, the pump or the internal portion of the cable is the culprit, an emergent and operative exchange is required. In these instances, profound cardiogenic shock can develop and temporary extracorporeal support might be necessary. The reported incidence of pump thrombosis in modern, continuous-flow devices ranges from 0.01 to 0.11 events per patient-year, but these figures likely represent underestimates as clinically silent thrombi often go undetected and not all devices are interrogated following explant (8–10). Factors, such as atrial fibrillation, sub-therapeutic systemic anticoagulation, and infection, have been associated with increases in device thrombosis.

Since 2011, the incidence of HeartMate II^®^ (Thoratec Corp., Pleasanton, CA) thrombosis has seen a dramatic increase from roughly 2 to 8% by January 2013 (9). Participating investigators theorized that this phenomenon was largely attributable to the “bearing-fibrin deposition theory”, which occurs in three distinct phases: (1) an initial phase characterized by hemolysis and thrombus deposition (termed “thrombus formation”); (2) “incomplete thrombus” resulting in red-cell destruction and abnormalities in LVAD performance; and (3) “complete thrombosis” culminating in device failure (9). While no unifiable factors were identified, flow reduction, whether due to arrhythmia, aortic regurgitation, or outflow obstruction, is an important element of the pathophysiology.

While there remains no consensus regarding the most sensitive and specific marker for early detection of imminent pump thrombosis, some have proposed a serum lactate dehydrogenase (LDH) level >4× normal (10) in combination with suspicious echocardiographic findings. While this value might capture patients with frank thrombosis, it might miss more subtle, earlier indicators of an impending problem at a time in which intervention might prove beneficial. Accordingly, LDH values >2.5× normal should prompt further interrogation with additional serum values [hemoglobin, haptoglobin, creatinine, total bilirubin, and international normalized ratio (INR)], chest X-ray, and a transesophageal echocardiogram. The latter can be utilized to evaluate the position of the cannula in respect to the ventricular walls and mitral valve. Additionally, if the aortic valve continues to open in response to incremental increases in the LVAD speed (ramp test), thus denoting incomplete emptying of the left ventricle, pump thrombosis should be suspected.

In the initial stages of device thrombosis, and assuming patient stability, non-operative management should be attempted. The patient should be started on a heparin infusion to halt propagation of the thrombus and to augment fibrinolysis. Active resuscitation should also be pursued, with or without force diuresis, to combat the detrimental effects of hemolysis on renal function. The patient should be closely monitored in a cardiac intensive care unit for signs of decompensation that might require urgent surgical management. In order to minimize the risk of pump thrombosis, patients should undergo a ramp study during their index hospitalization to provide a baseline for further evaluations. Anticoagulant and antiplatelet therapy should be implemented with an INR goal of 2–3.

In a large study that retrospectively examined approximately 1,200 devices, the rate of driveline fracture was 9.2% (11). A majority of these cases (87%) were isolated to the external portion of the cable and reinforcement was successful in most instances (76%) (11).

## Bleeding

Hemorrhage is one of the most common and challenging early and late complications associated with LVADs. The incidence of bleeding, whether requiring reoperation or red cell transfusion, is 0.16–2.45 events per patient-year (12, 13). The etiology of these events is multifactorial and includes: (1) management with systemic anticoagulation to diminish the risks of pump thrombosis and thromboembolic phenomena, (2) acquired von Willebrand Syndrome due to the non-physiologic, high-shear stress associated with the low-pulse pressures of continuous-flow devices (14, 15), and (3) the appearance of arteriovenous malformations (AVMs) (16, 17). While a majority of early episodes involves intrathoracic and/or mediastinal hemorrhage, the importance of these as causative factors diminishes over time. Conversely, the gastrointestinal (GI) tract is the principal source of late hemorrhage and, accordingly, a familiar effector of hospital readmission (14, 18). Several studies have demonstrated a growing rate of clinically relevant GI bleeds with the advent of continuous-flow devices (19, 20). While this association is most likely a combined result of manufacturer recommendations and guidelines of the HeartMate II Pivotal Trial to utilize antiplatelet medications and anticoagulants in LVAD patients to mitigate the up-regulation of the coagulation cascade, the observed rate was higher than would be attributable to systemic therapies alone (19).

Awareness of additional mechanisms that may contribute to this increased incidence of GI events has been the focus of several recent studies (14, 17). A sub-analysis of the HVAD BTT and CAP trials demonstrated a 15.4% incidence of GI bleeding with 86.1% of these events occurring >30 days from the initial operation (14). It is important to note that while the mean INRs were higher in those experiencing a bleed (2.4 vs. 1.6, *p* < 0.0001), a frank lesion, with an AVM being the most common, was identified as the cause of hemorrhage in 78% of the events (14). The pathophysiology associated with the development of these malformations is similar to the mechanism implicated in severe aortic stenosis (21). More specifically, a decrease in pulse pressure in these continuous-flow devices could result in relative splanchnic hypoperfusion and increased vessel friability. Another proposed theory to explain the high frequency of AVMs in this demographic involves the influence of increased intraluminal pressures, simultaneous vascular smooth muscle contractions, and subsequent relaxations of the bowel wall smooth muscle (16, 17).

In addition to adversely affecting vessel integrity, continuous-flow LVADs have been associated with acquired von Willebrand Syndrome (15, 20, 22). The biologic activity of von Willebrand factor (vWF) depends upon the cleavage of this molecule at a specific site by the ADAMTS13 metalloprotease and in order to promote normal coagulation, it is crucial to maintain equilibrium of the vWF multimer size. Studies have demonstrated that in situations of high-shear stress, the vWF multimers can undergo a conformational change, leading to an over-exposure of the cleavage domain. The shear forces on the artificial surfaces of these mechanical devices may deplete these larger multimers due to aberrant cleavage by ADAMTS13 and result in bleeding abnormalities (15, 20, 22).

Most GI bleeds require hospitalization, cessation of anticoagulation/antiplatelet therapies, institution of acid-reflux agents, and close hemodynamic and laboratory monitoring. Patients should be promptly evaluated by a Gastroenterologist for possible endoscopic management. Fortunately, several large studies have established that surgical intervention is rarely, if ever, a component of the treatment algorithm (14). These events are associated, however, with readmissions, non-device related infections, and subsequent thromboembolic complications (13, 14).

Several studies have supported a reduction in the target INR to 1.5–2.5 following a major GI bleed as most events are associated with an INR ≥ 2.5 (23). Unless the patient has had multiple life-threatening hemorrhagic events, complete cessation of anticoagulants and antiplatelet therapies is not recommended. In a meta-analysis evaluating risk factors associated with GI hemorrhage, nine of the studies included reported that a majority of the HeartMate II patients had therapeutic or sub-therapeutic INRs (24).

## Right Ventricular Dysfunction

Right ventricular dysfunction occurs in 9–44% of patients following LVAD implantation and is a significant contributor of short- and long-term morbidity and mortality (25–27). An initial examination into the comparative incidence of this condition in patients managed with pulsatile and continuous-flow device demonstrated no significant difference (28). Acute right heart failure is typically attributable to pre-LVAD organ system dysfunction [as indicated by the need for mechanical ventilation (29) or elevations in serum bilirubin and/or creatinine (30)] and/or marginal right ventricular function at the time of implant (27, 29).

A more insidious, chronic form of right ventricular dysfunction has also been identified, but its pathogenesis is poorly characterized. Multiple studies have demonstrated a leftward excursion of the intraventricular septum due to a reduction in left ventricular pressure and augmentation of right ventricular preload in patients supported by a LVAD (30, 31). These physiologic alterations are typically well tolerated and can even create a favorable pressure profile. In selected patients with marginal right ventricular function, however, even small variations in right-sided compliance can result in an inability to tolerate an increase in venous return. Conventional management strategies involve the assessment of flow through the device to normalize septal shift and the introduction of agents to minimize right ventricular afterload. The need for biventricular support in patients with severe right-sided impairment confers a considerably worse prognosis (27). In BTT patients with right ventricular dysfunction recalcitrant to medical management, heart transplantation should be strongly considered to avoid further deterioration of end-organ function.

## Infection

As per the INTERMACs Registry, LVAD-associated infections are categorized as follows: (1) *localized non-device infection* – infection involving an organ or region without evidence of systemic involvement; (2) *pump infection* – infection involving any component of the pump (pump or inflow or outflow cannulae); and (3) *percutaneous site and/or pocket* infection – positive culture from the tissue surrounding either the external portion of the driveline or the housing of the device. The latter has been largely attributed to trauma around the exit site that disrupts the tissue-line interface (32). In a large institutional series of 143 HeartMate II patients, driveline infections were diagnosed in approximately 12% of the population (33). The odds of developing an infection increased approximately 4% for each additional month of LVAD support (33). Progression to pocket or pump involvement was rare.

The incidence of driveline, pump pocket, or pump infection has diminished with the advent of newer generation, smaller axial-flow LVADs (e.g., driveline infections: 0.37 vs. 3.49 events per patient-year in HeartMate I and HeartMate II devices, respectively) (34). Furthermore, while the exact etiology is unknown, substantial reductions in infectious complications have also been appreciated in the recent era of continuous-flow implants (10.0 vs. 1.3 events per 100 patient-months, *p* < 0.001) (2). Most cases are successfully treated with antibiotic therapy and reinforcement of the exit site. More complex infections required surgical debridement in addition to medical management. Despite their varying clinical implications, these infections should not be trivialized as they are associated with costly readmissions and increased length of stay (32).

Patients should be educated on the importance of recognizing and reporting any traumatic events associated with the driveline as even small shear forces are capable of inciting an infectious complication. Some infections can be treated on an outpatient bases with a combination of intravenous and oral antibiotics that cover the most common pathogens, namely *Staphylococcus* species (32, 33). In patients who were present with noticeable cellulitis and other markers of infection, e.g., fever and leukocytosis, hospital admission and further workup are more appropriate. Blood cultures, aspirates for Gram stain and culture, and tissue samples should be collected if clinically indicated. If a deeper infection is suspected, abdominal ultrasound or CT imaging might be warranted.

If conservative management is not successful, then complete circumferential excision of all neo-epithelized tissue is required. Excision should continue until a tight adherence between the driveline and surrounding tissue is apparent. If the infection involves the pump pocket, external drainage can be attempted, but is often times unsuccessful. Surgical management of these infections can be difficult and multiple operations might be required to salvage the device.

## Aortic Valve Pathology

LVADs efficiently unload the left ventricle and diminish opening/closing of the aortic valve, which can result in progressive degeneration and a variety of valvular pathologies (35–37). This untoward consequence of chronic support can have deleterious effects on end-organ function and complicate cardiac recovery. The aortic valve is especially susceptible to acquired defects as its opening is entirely dependent upon forward flow from the left ventricle.

In a small series of patients with a mean LVAD time of approximately 1 year, commissural fusion was evident in all but one patient (38). The number of patients experiencing any aortic valve opening on echocardiogram diminished as the duration of support increased, with less than 50% having any valvular motion at 1 year (38). These morphological changes commonly resulted in mild to moderate central aortic regurgitation. Extrapolating from the heart failure population in which a diminished cardiac-output results in continued leaflet co-aptation, it has been postulated that continuous-flow LVADs create an environment of minimal-antegrade blood flow across the aortic valve (39). Furthermore, due to the placement of the outflow cannula in the ascending aorta, the closed valve is constantly exposed to a systolic pressure (40).

Device implantation can also result in a worsening of valvular competence, especially in patients with pre-existing aortic insufficiency (40, 41). Due to the small size of the outflow cannula relative to the native aorta, higher velocities are required to maintain physiologic flow rates (42). This can produce areas of high-shear stress and initiate a cascade of cellular events which culminates in aortic wall atrophy and sinus dilatation (37). These alterations might have a more profound effect in smaller patients with presumably narrower aortic diameters. Regardless, regurgitant flow can lead to further ventricular dilatation, increased pump work, and systemic hypoperfusion.

The clinical ramification of these valvular abnormalities is currently unknown. It is clear, however, that aortic valve disease in patients not destined for heart transplantation can lead to further deterioration in ventricular function and make device explantation less likely. Thus, there is a growing body of literature supporting ramp studies at the time of implant to ensure some aortic valve opening to reduce the long-term implications of prolonged valve closure (43). In LVAD candidates with baseline regurgitation or stenosis, concomitant aortic valve procedures should be considered. In cases of mild or worse regurgitation, a coaptive stitch can be attempted to regain valve competence. If the native valve cannot be repaired, a bio-prosthesis should be employed to restore proper valve mechanics.

## Thromboembolic Events

As previously mentioned, continuous-flow devices alter the equilibrium of the coagulation cascade and can predispose patients to thromboembolic phenomenon. It was initially believed that the blood–biomaterial interaction within the LVAD was the main stimulus for these findings (44), but surface modifications did not appear to alleviate the problem (45). More contemporary mechanisms involve the influence of the rotational flow of blood through the pump on thrombus formation. Additional patient-specific factors, including preoperative atrial fibrillation, have been shown to increase the risk for post-LVAD thromboembolic events (46).

While the true incidence of thromboembolic disease is hard to approximate given that confirmatory radiographic studies are typically only obtained in patients with frank neurologic impairment, series have estimated that this condition affects 10–17% of patients over the life time of their device (47, 48). These figures likely underestimate the true impact of LVADs on embolic formation as evident by a limited study which identified microembolic disease via transcranial Doppler in 20 out of 23 continuous-flow LVAD patients (45). The nidus of these thrombi is variable but the influence of aortic valve opening/closing on aortic root stagnation could emerge as an important factor in newer generation devices. To combat thrombus formation, most patients are administered a combination of anticoagulants and antiplatelet agents. Due to an increased risk of GI bleeding associated with greater periods of support, several studies have demonstrated acceptable thromboembolic protection with a more liberal anticoagulation strategy (49).

## Conclusion

Continuous-flow LVADs offer patients with advanced heart failure a viable option for prolonged support. As a growing number of patients are maintained on these devices for longer periods, the impact of late complications on outcomes will become increasingly more relevant. Providers should be aware of these potentially morbid events as prompt diagnosis and treatment are critical.

## Conflict of Interest Statement

The authors declare that the research was conducted in the absence of any commercial or financial relationships that could be construed as a potential conflict of interest.

## Abbreviations

BTT, bridge-to-transplant; DT, destination therapy, GI, gastrointestinal, LVAD, left ventricular assist devices, vWF: von Willebrand factor.
